# High-resolution imaging of the excised porcine heart at a whole-body 7 T MRI system using an 8Tx/16Rx pTx coil

**DOI:** 10.1007/s10334-023-01077-z

**Published:** 2023-04-07

**Authors:** Maxim Terekhov, Ibrahim A. Elabyad, David Lohr, Ulrich Hofmann, Laura M. Schreiber

**Affiliations:** 1 Comprehensive Heart Failure Center (CHFC), Department of Cardiovascular Imaging, University Hospital Würzburg, Am Schwarzenberg 15, 97078 Würzburg, Germany; 2 Department of Internal Medicine I / Cardiology, University Hospital Würzburg, Oberdürrbacher Straße 6, 97080 Würzburg, Germany

**Keywords:** Parallel transmit, RF-array, Excised heart, Ultra-high-field

## Abstract

**Introduction:**

MRI of excised hearts at ultra-high field strengths (${\mathrm{B}}_{0}$≥7 T) can provide high-resolution, high-fidelity ground truth data for biomedical studies, imaging science, and artificial intelligence. In this study, we demonstrate the capabilities of a custom-built, multiple-element transceiver array customized for high-resolution imaging of excised hearts.

**Method:**

A dedicated 16-element transceiver loop array was implemented for operation in parallel transmit (pTx) mode (8Tx/16Rx) of a clinical whole-body 7 T MRI system. The initial adjustment of the array was performed using full-wave 3D-electromagnetic simulation with subsequent final fine-tuning on the bench.

**Results:**

We report the results of testing the implemented array in tissue-mimicking liquid phantoms and excised porcine hearts. The array demonstrated high efficiency of parallel transmits characteristics enabling efficient pTX-based B_1_^+^-shimming.

**Conclusion:**

The receive sensitivity and parallel imaging capability of the dedicated coil were superior to that of a commercial 1Tx/32Rx head coil in both SNR and T_2_*-mapping. The array was successfully tested to acquire ultra-high-resolution (0.1 × 0.1 × 0.8 mm voxel) images of post-infarction scar tissue. High-resolution (isotropic 1.6 mm^3^ voxel) diffusion tensor imaging-based tractography provided high-resolution information about normal myocardial fiber orientation.

**Supplementary Information:**

The online version contains supplementary material available at 10.1007/s10334-023-01077-z.

## Introduction

Ultra-high-field (UHF) (${B}_{0}$ ≥ 7 T) MRI systems provide significant improvements in signal-to-noise ratio (SNR) compared to clinical systems at lower field strengths (e.g., ${B}_{0}$ ≤ 3 T). The effective RF wavelength in biological tissues at 7 T (${\lambda }_{\mathrm{eff}} \approx$ 12 cm) approaches dimensions of the human anatomy (e.g., head or thorax), which results in constructive and destructive interference patterns, and hence transmit ${B}_{1}$ field (${B}_{1}^{+}$) inhomogeneity [1, 2]. This problem can be addressed using a parallel transmit (pTx) RF amplifier system in combination with an optimized multi-channel coil array. Shaping of a uniform ${B}_{1}^{+}$ field distribution within a region-of-interest (ROI) enabled by manipulation of magnitudes and phases of driving voltages of each Tx channel and performed in a static [3–10] or dynamic manner [11, 12] is known as “B_1_^+^- “ or “RF-shimming”. Multi-channel Tx arrays, driven by the pTx system, have enabled applications in target regions, for example, the human head [1, 13], prostate [9], heart [11, 12, 14–21], and torso [22, 23]. A wide variety of different coil array designs, e.g., strip line elements [24, 25], local Tx/Rx loop arrays [26–31], or dipole antennas [17, 32–35], allowed for significant progress in body imaging at UHF.

In recent studies, the feasibility of cardiac MRI (cMRI) in pigs at a 7 T scanner using different dedicated 16-element transceiver coil arrays was proven [36, 37]. Furthermore, significant advancements in both ${B}_{1}^{+}$-shimming and parallel imaging capabilities were demonstrated for *in-vivo* cMRI in pigs using an antisymmetric transceiver 8Tx/16Rx loop array with L-shaped elements [38]. The same concept was successfully implemented in the design of 7 T human cardiac arrays [39]. Developed array designs enabled excellent parallel imaging characteristics with the capability of using high acceleration factors (up to *R* = 6) in 7 T cMRI. Finally, preliminary in-vivo results of the using antisymmetric concept of L-shaped elements for the high-density 8Tx/16Rx dipole array were reported [40].

MRI measurements of hearts *ex-vivo* at UHF can provide high-resolution ground truth data that complement *in-vivo* cMRI with image quality not compromised by physiologic motion. Moreover, scan times can be significantly longer than in animals or humans *in-vivo*. In extreme cases, scanning can be performed over many hours and, thus, can generate high-fidelity data for the assessment of morphological tissue properties like microstructure or tissue susceptibility effects [41–43]. For the highest SNR and spatial resolution, optimal transmit-and-receive characteristics are crucial factors. Available commercial coils for 7 T are usually designed for application in a specific body part (head, extremities) and may not always have optimal parameters for excised inner organs. In this study, we implemented a prototype of an 8Tx/16Rx transceiver array with antisymmetric L-shaped loop elements arranged on elliptically shaped housing and aimed to optimize a filling factor for an ultra-high-resolution 7 T MR-imaging of excised organs using a clinical whole-body scanner. The main goals were to validate the capabilities of the implemented array for (i) an efficient static parallel transmit B_1_^+^-shimming and (ii) parallel receive with high acceleration factors. The imaging performance of the new array was assessed, and pilot applications of high- and ultra-high spatial resolutions (up to 0.1 mm/pixel in-plane) multiparametric MR images were performed using excised porcine hearts.

## Methods

**Fig. 1:**
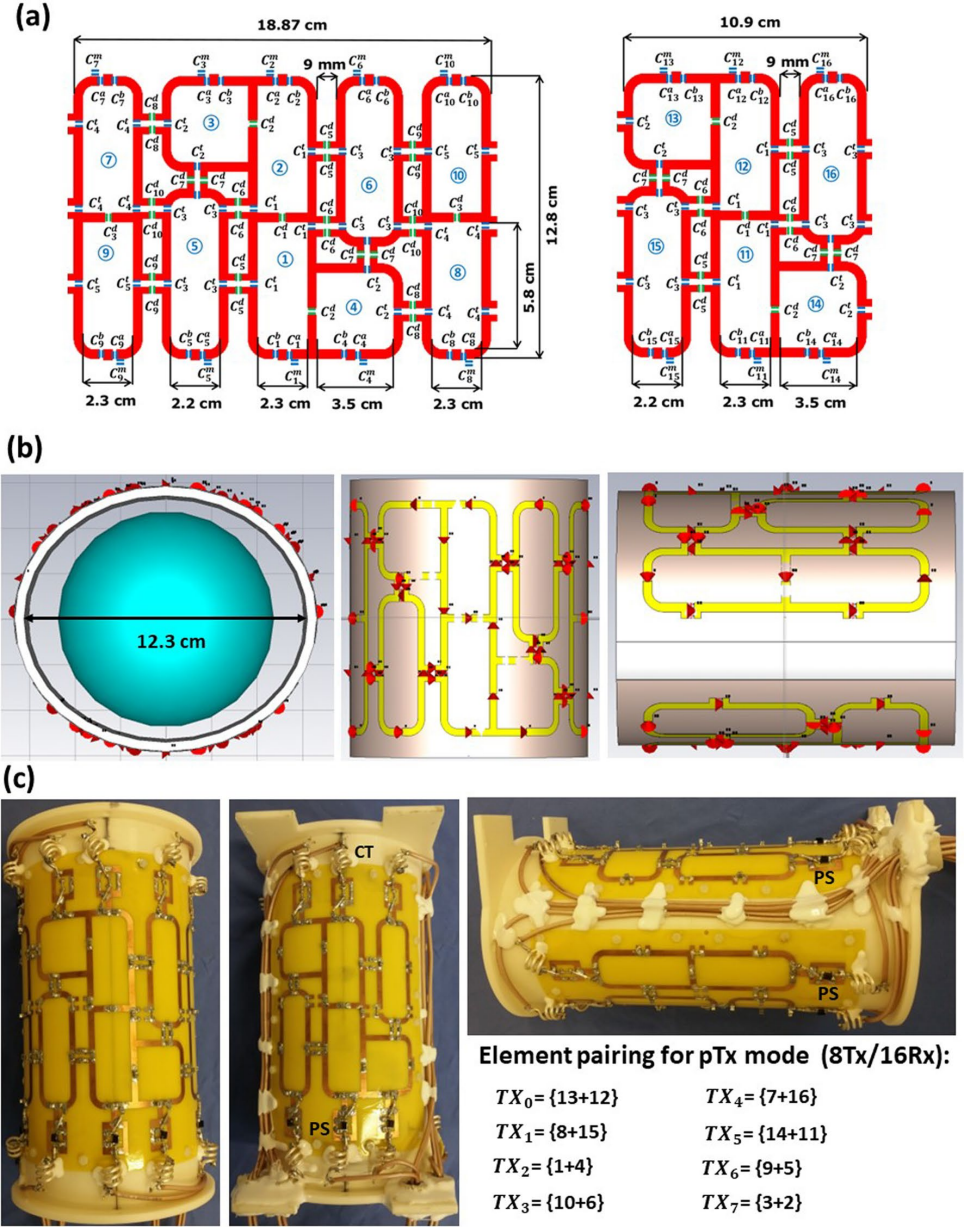
Design and schematic of the ex-vivo array for high-resolution imaging of excised porcine hearts. **a** Coil elements layout, dimensions, and element numbering. **b** RF simulation model of the array loaded with a 10-cm spherical phantom in front, top, and side views. **c** A prototype of the 16-element antisymmetric array in the top, bottom, and side views. Phase shifters (PS) and cable traps (CT) are labeled accordingly. Every two neighboring loops are paired to be interfaced to the corresponding Tx channel of RFPA (channel numbering is according to the MRI system notation) to form an 8Tx/16Rx array configuration

The array is designed as a printed circuit board (PCB) with 16 loop elements bent around an elliptically shaped housing with major/minor axes of 12.3/10.7 cm (Fig. 1a,b). The elements were etched using 0.3 mm Cu trace of 4 mm width printed on a 0.3 mm FR4 substrate (${\upvarepsilon }_{\mathrm{r}}$ = 4.24 and $\mathrm{tan\delta }$ = 0.014 at 297.2 MHz). PCB was manufactured by Q-print Electronic GmbH (Heddesheim, Germany). The array was split into two sections to keep 5.4 cm spacing on the housing for routing the RF cables (Fig. 1c). The top and bottom sections comprised 10 and 6 elements, respectively, as shown in Fig. 1a. The dimensions of elements 1, 2, 7, 9, 11, 12 = 2.3 × 5.8 cm^2^, elements 3, 4, 13, 14 = 3.5 × 3.5 cm^2^, and elements 5, 6, 15, 16 = 2.2 × 6.9 cm^2^. The decoupling between the central elements (1&2) and (11&12) was accomplished using a common conductor and shared decoupling capacitors (SDC) (C_1_^d^). The elements (3,4,5,6) and (13,14,15,16) were distributed in an antisymmetric manner around the central loops of the top and bottom array sections, respectively. Elements 3 and 4 were decoupled with elements 1 and 2 using SDC (C_2_^d^). Elements 5, 7, and 9, and the identical elements 6, 8, and 10 were decoupled from the neighboring elements using capacitive decoupling (C_5_^d^, C_6_^d^, C_7_^d^, C_8_^d^, and C_9_^d^) using a decoupling gap of 9 mm. The total external dimensions for the top and bottom PCB parts were 18.9 × 12.8 cm^2^ and 18.9 × 10.9 cm^2^, respectively. To form an 8Tx/16Rx array compatible with the MRI system’s 8 TX-channel RF Power Amplifier (RFPA), every two neighboring elements were combined in one Tx channel (Fig. 1c). The array was connected to a 16-channel interface with 16-Tx/Rx switches and 16-preamplifiers via four ODU plugs (ODU GmbH & Co. KG, Muehldorf, Germany). To adjust the default hardware-implemented phase shifts between the 16 coil elements, a discrete low-pass π-network phase shifter (PS) consisting of two equal capacitors and one non-magnetic inductor (Coilcraft, Inc., Silver Lake Road, Cary, IL, USA) was designed. These phase shifters were inserted between the coil elements feeding ports and the cable traps (CT) as shown in Fig. 1c.

### A. EMF Simulations

EMF simulations were carried out using CST-MICROWAVE-STUDIO (CST-MWS, Dassault Systèmes SE, France) time-domain solver (transient solver), which is based on the finite integration technique (FIT). The CST mesh parameters were: 70 lines per wavelength, a lower mesh limit of 70, and a mesh line ratio limit of 80. A local mesh setting with an isotropic step width of 1 mm and an edge refinement factor of 4 was set for the curved Cu layouts. Open (add space) boundary condition was used. RF-circuit co-simulation [44] was employed in CST-Design-Studio (CST-DS) for matching and tuning to obtain an initial guess for the optimal lumped elements.

### B. Interfacing of the array to a pTx RFPA

To interface the designed array to 8 Tx channels of RFPA, a pairing of elements was performed based on previous experience of designing arrays for pig and human MRI with L-shaped antisymmetricaly arranged elements [36, 37, 45]. For example, the elements (2&3) form an L-shaped pair connected to the RFPA-channel *TX*_*0*_ (Fig. 1a, c). The same principle was used for the channels *TX*_*2,*_* TX*_*5,*_ and *TX*_*7*_. For the other elements, pairing the proximity between the nearest elements, and the shortest path to route the RF cables to the interface were taken into account. The complete pairing list is shown in Fig. 1c. The array was connected to the 8Tx/16Rx interface with 16-Tx/Rx switches and 16-preamplifiers (Rapid Biomedical GmbH, Rimpar, Germany) via four plugs (ODU GmbH & Co. KG, Mühldorf a. Inn, Germany). The detailed scheme of interfacing can be found in supplementary materials of the work [40]. Each RFPA channel provides up to 2 kW peak power at the input interface plugs. The length of the feeding cables (between cable traps and interface sockets) is ~ 45 cm for one pair of plugs and ~ 25 cm for another pair.

### C. Customer pTx-based ${B}_{1}^{+}$ shimming

The customer B_1_^+^ shimming procedure was implemented based on the so-called “relative” ${B}_{1}$-maps. These maps were reconstructed from complex GRE images acquired by driving individually each Tx-channel of the array and acquiring signal received simultaneously by all elements. It was demonstrated that such B_1_^+^ maps can be efficiently employed as a proxy of the absolute (flip-angle) ${B}_{1}^{+}$ maps to perform B_1_^+^ shimming in UHF MRI. A detailed description of the acquisition and reconstruction of relative B_1_^+^-maps used in both static and dynamic pTX-shimming procedures can be found in [11, 15, 46]. A brief mathematical description of the optimization problem and two cost functions used to promote the “B_1_^+^ homogeneity” and “B_1_^+^-efficiency”, respectively, are provided in Appendix 1. The optimization was performed within the manually defined 3D volumes. For the homogeneous spherical phantom, the optimization volume was a cylinder with ~ 75 mm diameter and 50 mm height (as shown in Fig. 3). For the B_1_^+^-shimming in the excised heart sample, the optimization region was a rectangular slab with dimensions 60×60 x 60mm positioned to cover mid-myocardium and apical areas. The computed complex components of the array driving voltages for the individual Tx channels were set to the RFPA using B_1_^+^ adjustment platform of the MR scanner. To ensure a fair comparison between different pTX-shimming settings regarding transmit efficiency, the complex vectors of transmit voltages were normalized to have the unity norm (Appendix 1).

### D. Sample preparation

Following the “3R” principle for testing the new array performance, we have used the excised hearts kindly provided after the authorized use of animals in other studies. The *n* = 3 intact hearts of healthy piglets were obtained from project 55.2 2532–2-256 (District of Low Franconia, Germany). The heart of ~ 80 kg pig euthanized approximately 60 days after myocardial infarction, which was obtained from the study 55.2 DMS 2532–1134-16 (District of Low Franconia, Germany). The infarction was induced by 90 min balloon catheter occlusion of the left anterior descending coronary artery, followed by reperfusion. For the healthy pigs' hearts following the excision, the atria of the hearts was removed to ease the release of trapped air. Fixation was achieved via immersion in 10% neutral buffered formalin within 3 h of cardiac arrest. Hearts were placed in a plastic container and the sample position was fixed using sponges. The container was then slowly filled with Fomblin™ oil and excess air was removed from the sponges and the heart using a vacuum desiccator. Additionally, for B_1_^+^-shimming and coil characterization measurements, the homogeneous phantom was used. The phantom was implemented using a 100 mm diameter acryl glass sphere filled with the polyvinylpyrrolidone (PVP) water solution mimicking the electric properties of biological tissue [38].

### E. MRI measurements

All measurements were performed using a whole-body 7 T MRI system (Magnetom™ “Terra”) with 8-channel parallel transmit (pTx) system, (Siemens Healthineers, Erlangen, Germany). The characterization of receiving sensitivity and parallel imaging capabilities of the new array was done by comparison with the commercial 1Tx/32Rx head coil (Nova Medical, USA).

Array characterization for parallel receiving was performed using a dedicated vendor-supplied protocol (named “coil utility” on the Siemens MRI system). This protocol provides data acquisition using a fast spoiled GRE pulse sequence and reconstruction of SNR maps, g-factor maps, and noise correlation matrices. Imaging parameters were TR/TE = 9/3.8 ms, FA = 10^0^, voxel size = 0.4 × 0.4x5mm, and matrix = 256 × 256. Measurements were performed in the described above homogenous spherical phantom and excised porcine hearts (*n* = 3). To characterize and compare the receive sensitivity of the head coil and designed ex-vivo array, we used SNR maps reconstructed by the above-mentioned protocol. The SNR maps normalized using absolute B_1_^+^ maps are further referred to as receive sensitivity (RxS).

The absolute B_1_^+^ maps were acquired and reconstructed using the vendor-provided sequence “tfl_rfmap” based on the saturation-prepared turbo-FLASH pulse sequence [47]. For the off-line B_1_^+^ optimization, the relative B_1_ + maps were acquired and reconstructed using the customized GRE sequence as described in works [9, 14, 15]. For both absolute and relative B_1_^+^ mapping, a fixed reference transmitter voltage V_ref_ = 100 V was set via the scanner adjustment platform. To visualize the effect of B_1_^+^-shimming in the volume, 3D GRE images of the spherical phantom were acquired with TE/TR = 3.2/20 ms, FA = 10^0^, and voxel size = 1 × 1 ×  2 mm.

T_2_* maps with 1 mm isotropic resolution were generated from 2D gradient multi-echo sequence (mGRE) data acquired with both coils for the same heart sample. Measurement parameters were: number of averages NA = 32, TR = 800 ms, 9 echoes per excitation with TE = [2.5.0.18.7]ms. To compare the parallel imaging capabilities of both coils, the mGRE data were acquired using consequently increasing the GRAPPA acceleration factor *R* = [2.0.6].

To assess the performance of the coil in ultra-high-resolution imaging of myocardial scar tissue after infarction, imaging was performed using a turbo-spin-echo sequence with the following parameters: echo train length = 4, TE/TR = 15/2000 ms, acquisition matrix = 960 × 810, slice thickness = 1.0 mm and 0.8 mm, physical pixel size in-plane = 0.1 × 0.1 mm, number of averages = 16, and acquisition time 42 min.

Diffusion tensor imaging (DTI) was performed using a spin echo diffusion sequence with Stejskal-Tanner diffusion encoding and EPI readout. To acquire a whole-heart DTI dataset with an isotropic spatial resolution of 1.6 mm, the vendor “epi_diff” pulse sequence was used with the following parameters: TE = 56 ms, acceleration factor *R* = 3, 30 diffusion directions after Skare[48] (b = 2000s/mm^2^), frequency bandwidth = 1414 Hz/pixel, and 50 averages. DTI data processing and fiber-tracking reconstruction were done using DSI Studio[49] and Matlab (MathWorks, Natick, USA).

## Results

**Fig. 2:**
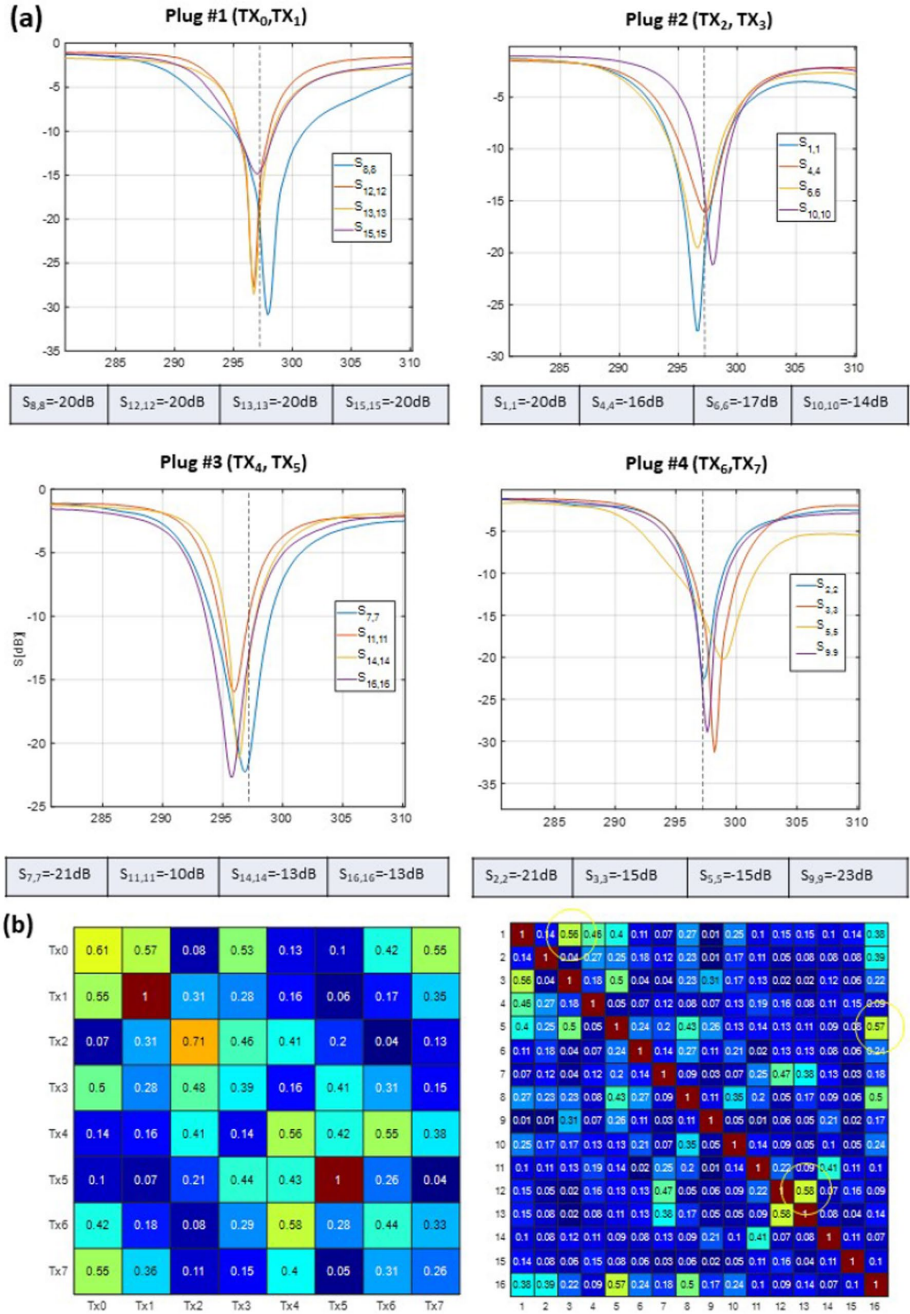
**a** Measured S-parameters versus frequency for the four ODU plugs when the array was loaded with a 10-cm spherical phantom (${\upvarepsilon }_{\mathrm{r}}$= 59.3 and $\upsigma$=0.79 S/m). Most of the elements were matched better than − 14 dB. Only elements 11 and 16 were matched to − 10 and − 13 dB. **b** The noise correlation coefficient matrix for 16 receive elements and normalized scattering matrix for 8 Tx channels measured by the MR scanner. Yellow circles on the noise correlation matrix show pairs with relatively high transmission coefficients (> -9 dB) leading to increased noise correlation

Figure 2a shows measured S-parameter curves measured by VNA for the 16-element dedicated array versus frequency for the four ODU plugs when the array is loaded with a 10-cm spherical phantom filled with PVP solution (${\upvarepsilon }_{\mathrm{r}}$   = 59.3 and $\upsigma$ = 0.79 S/m). Most of the elements were matched better than − 14 dB. Only elements 11 and 16 were matched to − 10 and − 13 dB. All 16 scattering curves demonstrate clean frequency profiles without splitting which would be evidence of insufficient element’s decoupling. Figure 2b (left) shows S-matrix for 8 Tx channels measured by the scanner directional coupler (DICO) and saved by the RF-safety watchdog system. Figure 2b (right) demonstrates the noise correlation coefficient matrix. A high noise correlation (> 0.5) is observed for 3 pairs of elements which are related to the high transmission coefficients (e.g., S_12,13_ =− 8 dB as measured by VNA). The increased transmission coefficient as well as uneven reflection coefficients in the 8-Tx S-matrix measured by the scanner DICO may be related to the inhomogeneous loading of the coil elements by the 10 cm spherical phantom.

**Fig. 3:**
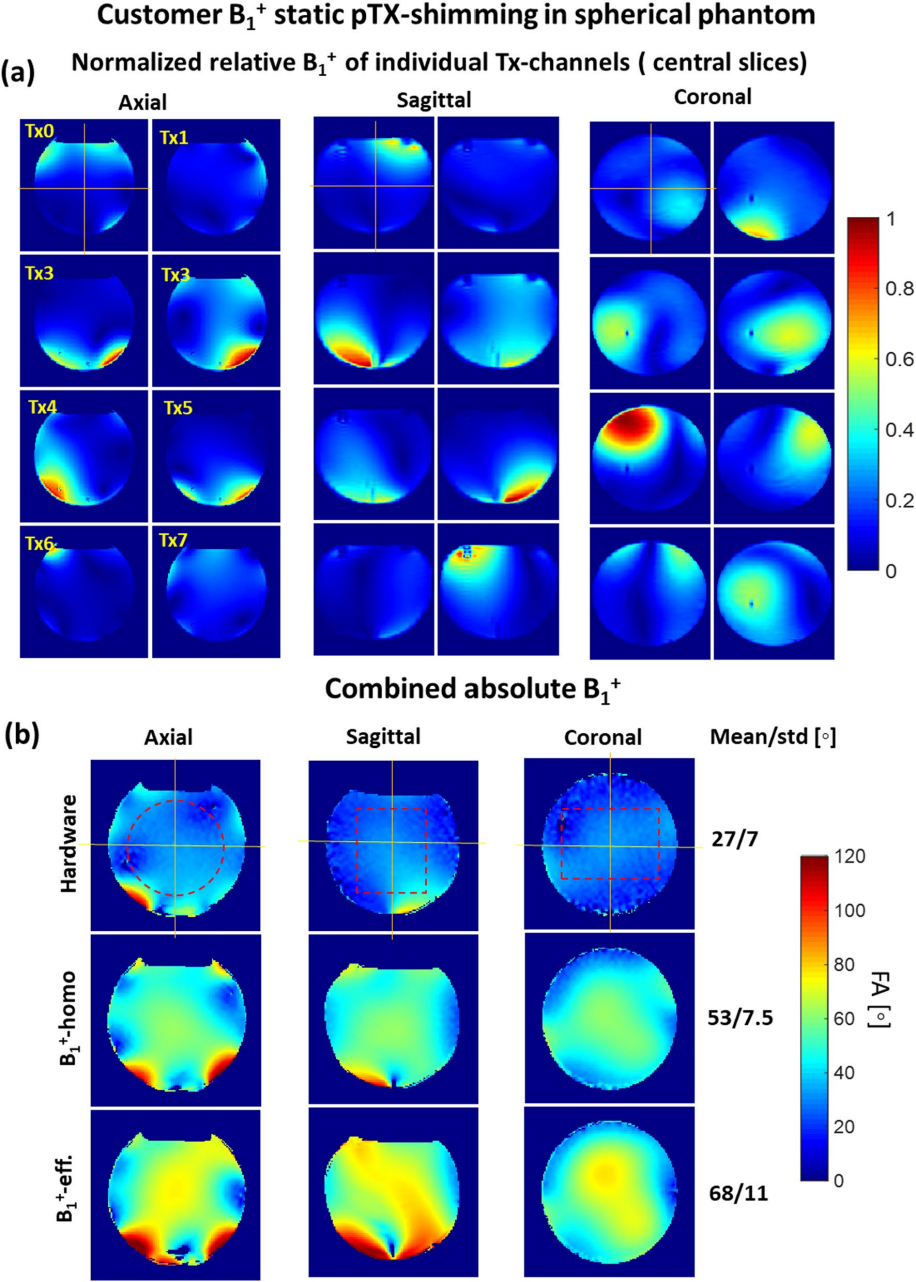
Static pTX-based B1 + shimming in the homogeneous spherical phantom based on the customer optimization procedure (Appendix 1). (**a**) Relative normalized B1 + maps of the individual channels used for B1 + optimization. Central slice projections (solid lines) are shown. (**b**) Combined absolute B1 + maps acquired with default array phasing ("Hardware") and using pTX-based RF-shimming settings computed within the labeled ROI (dashed red lines) by optimization of “B1 + homogeneity” and “B1 + efficiency” cost functions (Appendix 1). All three combined B1 + maps are acquired with the same transmitter reference voltage of 100 V. The mean and standard deviation of the flip angle within shimming ROI are shown for each shimming setting

Figure 3a demonstrates three central slices from the 3D relative B_1_^+^ maps acquired in the spherical phantom. These maps were used for the validation of the pTX-based B_1_^+^-shimming capability of the array. One can observe that the localizations of the regions with maximal intensity in the B_1_^+^ profiles of the individual Tx channels are well separated which is a prerequisite for an efficient static B_1_^+^-shimming. Absolute B_1_^+^ maps for the same slices are shown in the Supplementary Materials (Figure S1). Panel 3b shows absolute combined B_1_^+^-maps before and after RF-shimming performed using cost functions optimizing B_1_^+^-homogeneity and B_1_^+^-efficiency represented, respectively, by Eq. (2) and Eq. (3) of Appendix 1. The optimization using the former cost function improved the coefficient-of-variation of the flip-angle within the optimized volume by about factor 2, whereas using the latter cost function increased the mean flip-angle value by 150%. Supplementary material Figure S2 shows the effect of using the computed optimized B_1_^+^-shimming settings on the acquired GRE images. Using the same RF-pulse voltages, the signal intensities within the optimization volume were increased by factor > 2 by applying both optimized RFPA voltages settings for the B_1_^+^ shimming.

**Fig. 4:**
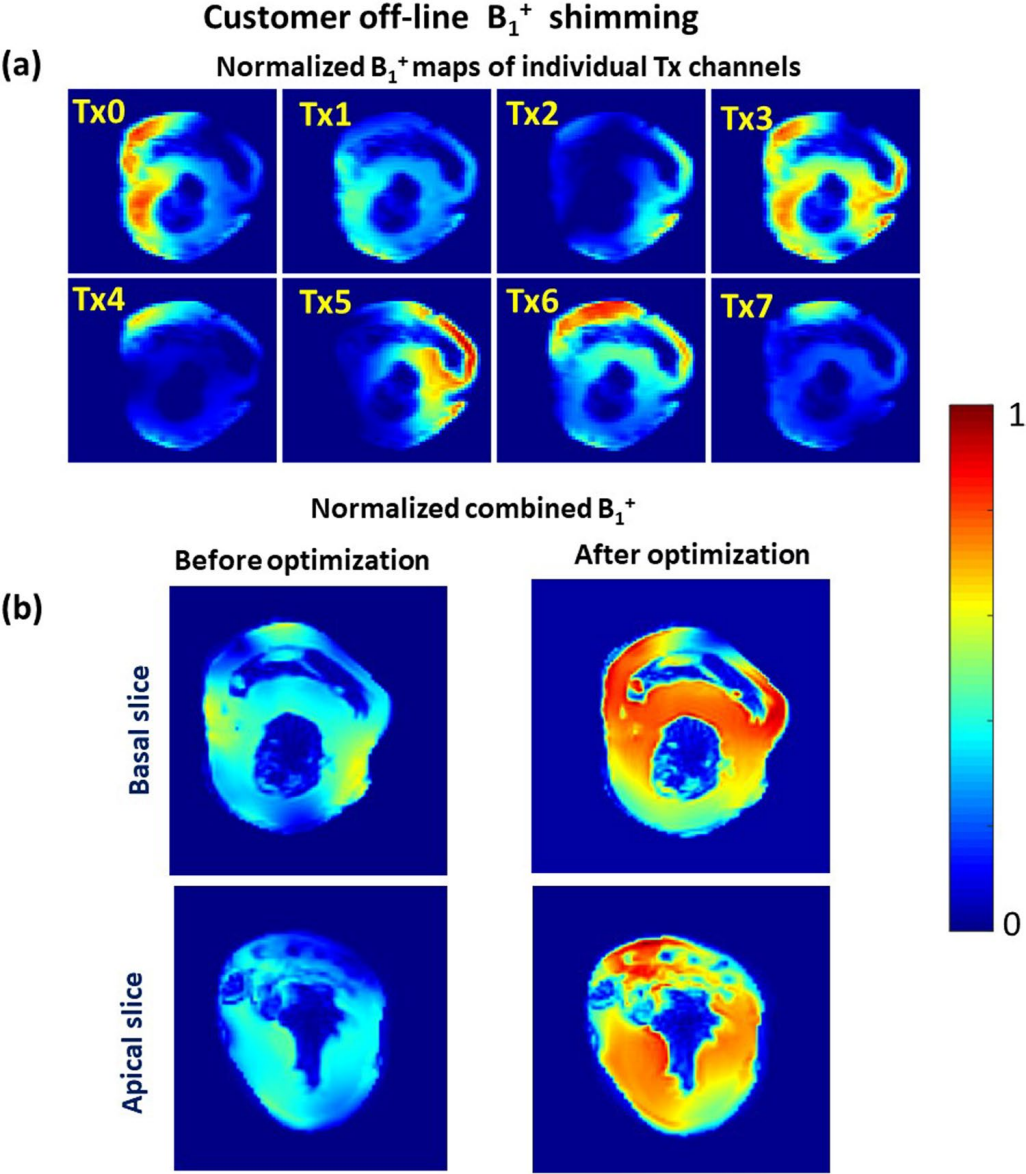
Customer B1 + shimming for the excised heart sample. (**a**) Normalized relative B1 + -maps of the eight individual transmit channels (one axial slice shown). (**b**) Normalized combined relative B1-maps before pTx optimization (with hardware phases) and after pTx-based RF-shimming

Figure 4a,b shows the individual relative B_1_^+^ maps and the resulting combined B_1_^+^ field in the excised heart sample before and after the B_1_^+^ shimming targeted for the B_1_^+^-efficiency. Despite the size of the excised heart being relatively small compared to the spherical phantom, the peaks of B_1_^+^ profiles of most of the individual TX channels are well localized within the sample. Therefore, the optimization procedure is capable to increase significantly both homogeneity and the mean value of the combined B_1_^+^.

**Fig. 5:**
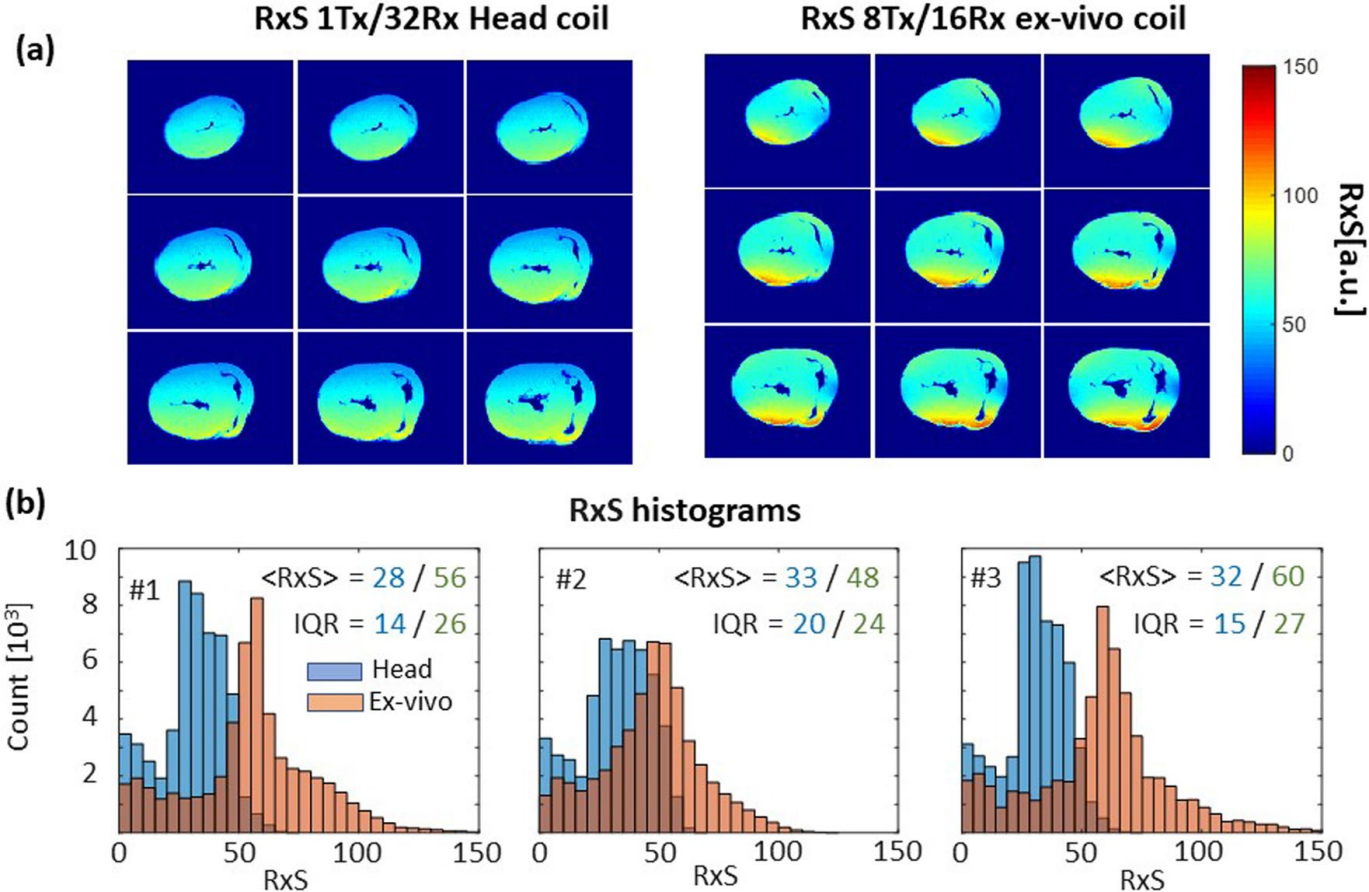
Rx-sensitivity comparison for head coil and ex-vivo coil. (**a**) Example of Rx-sensitivity maps (9 slices of 32) acquired using ex-vivo array in the heart sample #3 in comparison to the same slices acquired with 1Tx/32Rx head coil. A higher mean value of normalized SNR is observed visually for the ex-vivo array. **b** Histograms of Rx-sensitivity for 3 ex-vivo heart samples for the head coil (blue bars) and ex-vivo array (red bars). The advantage of the ex-vivo array in filling factor manifests in essentially higher mean Rx-sensitivity. The larger heterogeneity (characterized by interquartile range) of normalized SNR is, however, a consequence of smaller Tx/Rx ex-vivo array volume compared to the resonator of the head coil

Figure 5a, b shows a comparison of Rx-sensitivity maps and histograms for both the 1Tx/32Rx head coil and the new 8Tx/16Rx array. Whereas homogeneity of the RxS (IQR metric) is higher for the head coil with a large volume resonator and essentially larger size of Rx array, we found that Rx-sensitivity is up to factor 2 times higher for the new ex-vivo array because of the optimal filling factor.

**Fig. 6:**
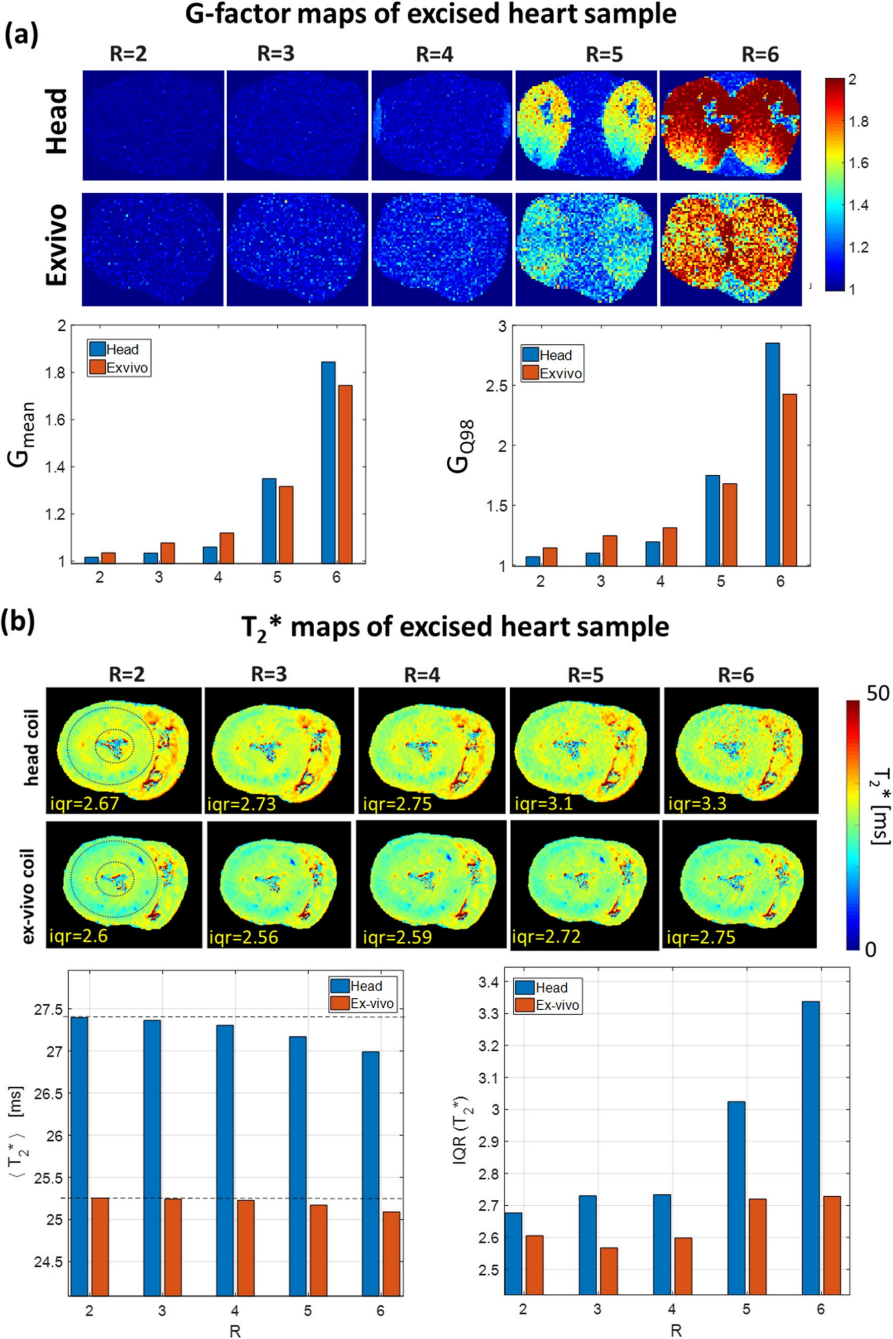
Noise amplification in parallel acquisition imaging. (**a**) Maps of the g-factor and statistical metrics for both coils measured in the excised heart sample #3. The mean and maximal (98-percentile) g-factor values become higher for the head coil for the high acceleration factors *R* = 5,6. **b** T2* maps of heart sample #3, reconstructed from mGRE images using increasing GRAPPA acceleration factors. There is a remarkable increase of the noise amplification at (*R* = 5,6) for the head coil leading to 20% increase T2* IQR values within the marked (dashed line) myocardial region when compared with results obtained with the new excised tissue coil

Figure 6a shows the g-factor maps of the 1Tx/32Rx head coil and the 8Tx/16Rx ex-vivo array in the mid-myocardium slice of the excised heart sample. Despite the difference by a factor of two in the number of receiving elements of both coils the statistical metrics of the g-factor (mean value and 98-percentile) are very similar at acceleration factors *R* = 2,3 and 4. For the higher acceleration factors (*R* = 5 and 6), these metrics are lower for the new ex-vivo array compared to the head coil.

Figure 6b shows a representative slice of the T_2_^*^ maps in an excised heart acquired with both coils using GRAPPA parallel receive acceleration factors between 2 and 6. One can observe that at high acceleration factors *R* = 4, 5, and 6, the T_2_* maps acquired with the head coil show more “salt-pepper”-like noise compared to the maps acquired with the new array. Accordingly, the heterogeneity of T_2_^*^ values, characterized by the interquartile range, increases for the head coil more significantly (up to 20% at *R* = 5 and 6) than that for the new array.

**Fig. 7:**
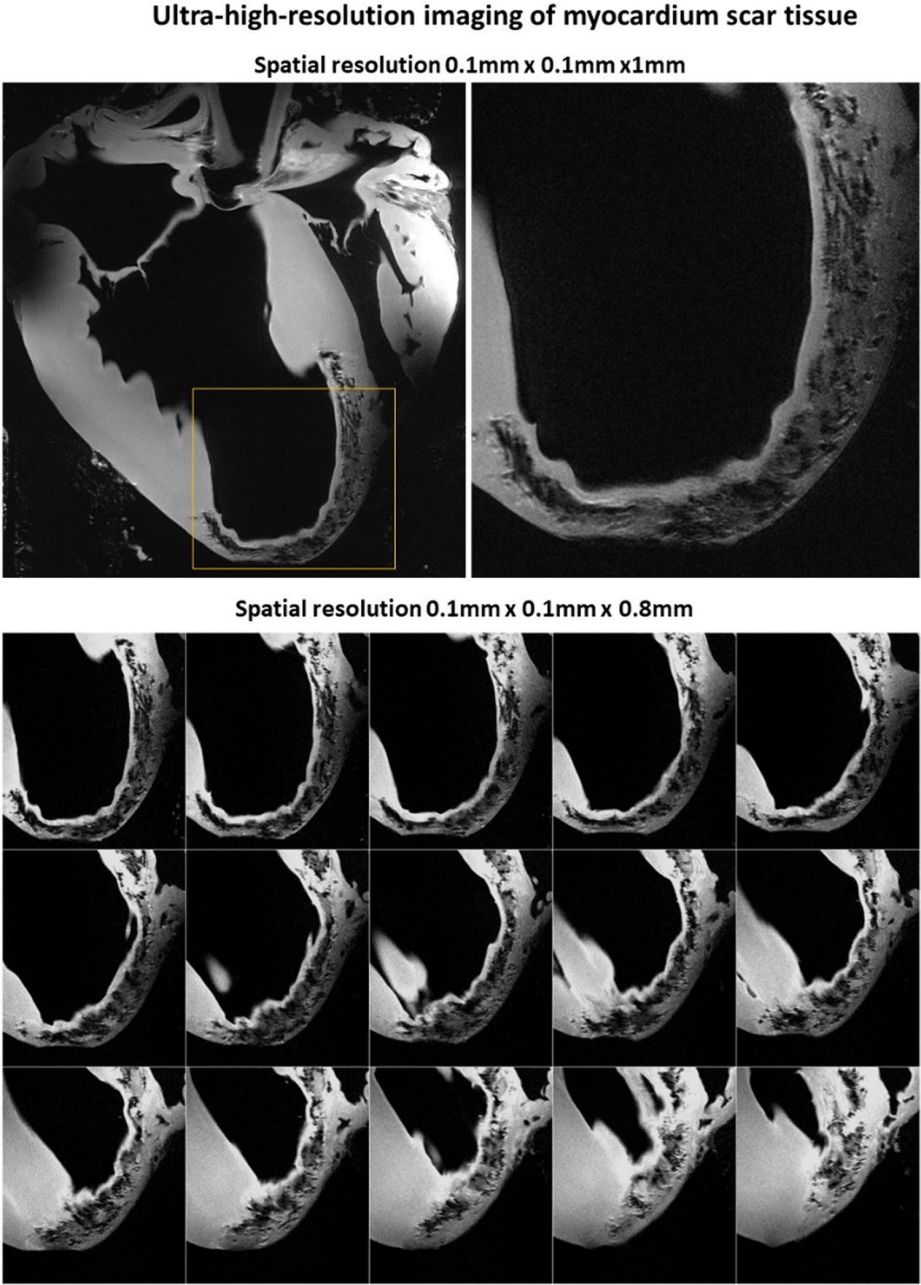
Ultra-high spatial resolution images of an excised heart 60 days after infarction. The top row shows a single slice with a whole-heart long-axis view and zoomed region of the scar (labeled by the yellow rectangle). The bottom row shows zoomed regions of individual slices covering the scar region. Images were acquired with parallel receive acceleration factors *R* = 2 (top row slice thickness 1 mm, acquisition time ~ 55 min) and *R* = 3 (bottom row, slice thickness 0.8 mm, acquisition time ~ 40 min), respectively. In both cases, a very high level of detail is observed in post-infarction scar tissue

Figure 7a, b shows ultra-high-resolution images of scar tissue in the excised heart. The images demonstrate a very high level of detail in both heart anatomy (a) and especially scar tissue structure within the myocardial wall. Sub-millimeter slice thickness allows for resolving the 3D network of the scar within the myocardial wall including the structure of scar edges (peri-infarct area). This transition zone between scar and intact tissue is especially important diagnostically for identifying the risk of malignant arrhythmias and sudden cardiac death [50].

**Fig. 8:**
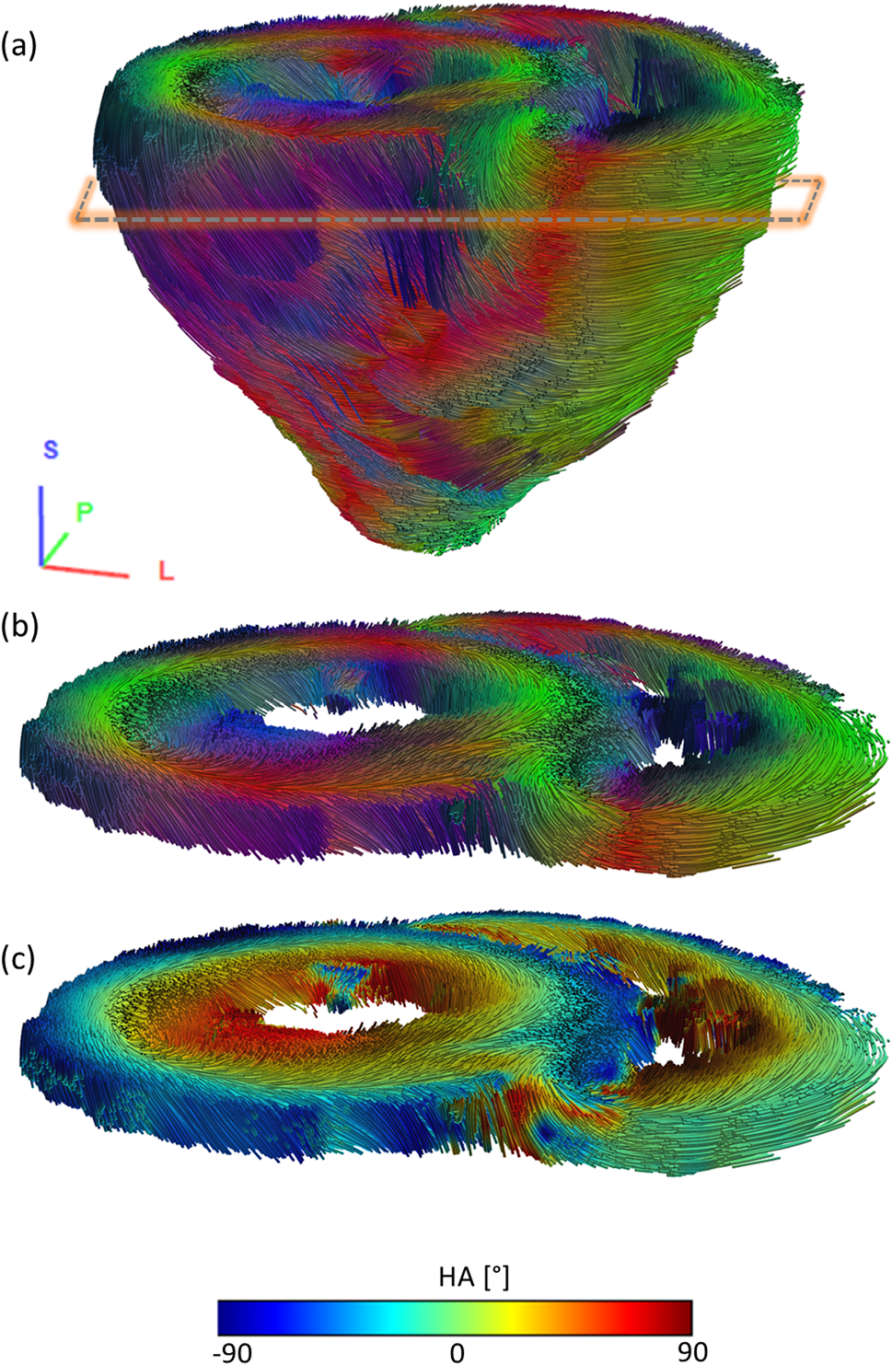
Tractography of myofiber bundles based on high-resolution DTI imaging of a fixed porcine heart acquired using the new coil. **a** Tractography showing 30,000 fiber bundle tracts visualized using tubes with a thickness of 5% of the voxel size. Thresholds for tracking were FA: 0.1, min bundle lengths: 10 mm, max bundle lengths: 300 mm, step size: 0.5 of the voxel size, and angle: 60°. Color-coding of the tracts corresponds to the main eigenvector orientation. A respective coordinate system is given in the bottom left. The dashed box approximates the position of the slab shown in (**b**) and (**c**). (**b**) Excerpt (thickness corresponds to three slices) from the whole-heart tractography. The high resolution enables the visualization and assessment of papillary muscle (dark blue) within the left and right ventricle or the transmural helical configuration of myocyte bundles as well as the intersection of the left and right ventricle with a fidelity unobtainable in-vivo. Color-coding of the tracts corresponds to the main eigenvector orientation as in (**a**). (**c**) The same tractography excerpt with color-coding of the tracts corresponds to the local helix angle value

Figure 8 shows representative high-resolution tractography visualizing distinct structures of myocardial fiber orientation in the intact heart. High isotropic spatial resolution enabled detailed visualization of papillary muscle (dark blue), the intersection of the left and right ventricle (panel b), and the helical configuration of myocyte bundles with a transmural variation of the helix angle (panel c). The further DTI results presenting fractional anisotropy, principal eigenvalues, and helix angle and E2A angle maps are shown in the Supplementary Materials (Figure S3).

## Discussion

Due to a lack of commercially available coils for imaging excised tissue, most researchers typically use standard RF arrays designed for clinical applications in humans. We demonstrated the concept of the inexpensive dedicated array for UHF-MR-imaging of excised hearts based on 16 loop elements with an antisymmetric L-shaped arrangement used for both parallel transmit-and-receive. The unitary architecture based on the single printed PCB requires moderate hardware and manpower resources for implementation in comparison to the coils using separate transmit-and-receive parts. The comparison of the new excised tissue array with the conventional head coil provided remarkable insights. Despite the small dimensions of the dedicated array targeted to provide a high filling factor for an excised heart, a static pTX RF-shimming allowed for significant (by factor > 2) improvement of B_1_^+^ homogeneity and efficiency. This is an interesting finding in such a relatively small coil, because, typically, pTx techniques at 7 T are applied for B_1_^+^ shimming in relatively large objects. A higher filling factor of the new array provided a factor two increased Rx-sensitivity when compared with the 1Tx/32Rx channel head coil.

Results in parallel receive mode tended to be interesting. Despite two times fewer amount of receive elements, the g-factor of the new array was found to be superior to that of the head coil 32Rx-only array, especially for the high acceleration factor *R* > 4. This is probably an effect of optimal dimensions and geometry. Remarkably less “salt-pepper”-like noise GRAPPA reconstruction artifacts were observed in T_2_^*^ maps reconstructed from images acquired with acceleration *R* = 4.0.6 using the new 8Tx/16Rx transceiver array in comparison to the commercial 1Tx/32Rx head coil with separated Tx-resonator and Rx array.

The coil demonstrated outstanding potential to perform post-mortem non-invasive “MRI-histology”-similar images of the heart both at sub-millimeter (100 µm) level using ultra-high-resolution multiparametric imaging (proton density, T_2_, T_2_^*^) and on the microscopic level using diffusion-based MRI tractography of the cardiac muscles. Future applications in diffusion imaging will greatly benefit from the low noise amplification and a minimal level of reconstruction artifacts even at high parallel imaging acceleration. The latter is of high importance for DTI application at 7 T because of the possibility to reduce echo time in EPI readout and, thus, to minimize SNR losses due to short T_2_* at 7 T, in particular, in fixed hearts [41] or other specimens. The resulting high image resolution (≤ 1.6 mm isotropic) will enable the assessment of anatomical regions previously hardly accessible, e.g., the intersection of the left and right ventricle or the right ventricle [51]. Overall, ex-vivo imaging in general, and excised tissue imaging in particular, provide valuable supplementary information and enforce a close linkage between *in-vivo* MRI and optical microscopic data for histology. Moreover, such MRI data may become important as high-fidelity ground truth in the development of machine and deep learning applications, in situations where such data cannot be acquired in-vivo or where in-vivo acquisitions are not feasible because of patient or animal constraints.

Furthermore, the dedicated array for excised tissue imaging is helpful in studies on the validation of new MRI applications. Potentially, the dedicated coil for MRI of the excised heart can be used in large-animal adapted Langendorf-type models [52, 53] Another potential research field may be human hearts after excision due to cardiac transplantation or in xenotransplantation research. The mentioned beating hearts may also be studied by extension of the coil with a dedicated organ chamber and perfusion system. Further fields of in-vivo application may be preclinical imaging of middle-size small animals (e.g., guinea pigs, rats, and small rabbits) performed in the clinical scanner or human imaging of the wrist or elbow.

The drawback of the compact size of the new array is the proximity effect of relatively small loop elements leading to the signal inhomogeneity near the array surface and requiring additional efforts for the normalization of signal intensities. It puts additional demands on the fidelity of the MRI system’s receive chain, which imposes no problem with today’s scanners' receive systems' dynamic range, and with adequate adjustment of the receive chain for the object under investigation. Another limitation of the study was the use of the Fomblin™ environment for the allocation of the pig hearts used in this study. Fomblin™ has a low electric permittivity that leads to a less sophisticated standing wave pattern with the array volume. However, submersing tissue in the synthetic oil may lead to alterations in the biological properties of the tissue [41] and would not allow experiments with perfused hearts. As an alternative, fresh non-fixed excised hearts could be placed within an isotonic NaCl–water solution with high permittivity ( ε = 79) which is essentially more challenging for the B_1_^+^-shimming. A second limitation was using only a static pTX B_1_^+^-shimming in all studies. Further improvement of SNR and its homogeneity may be achieved using dynamic pTX B_1_^+^ -shimming with tailored [12] or universal pTX pulses [11].

## Conclusion

We presented the novel concept of a dedicated array for ultra-high-resolution MRI of excised pig hearts on a clinical whole-body MRI system. The prominent features of the array are (i) the 8TX/16Rx architecture based on 16 anti-symmetrically allocated loops for the parallel transmit-and-receive combined with (ii) the dimensions optimized for the small targeted sample volume. Up to factor 2 gain of receive sensitivity in comparison to the commercial 1 T/32Rx head coil was achieved in the pilot study. The capability to provide an efficient ${B}_{1}^{+}$ shimming based on the pTX platform and, at the same time, ensuring a robust parallel imaging capability for high- and ultra-high-resolution multiparametric MRI of excised pig hearts were demonstrated.

### Electronic supplementary material

Below is the link to the electronic supplementary material.

Supplementary file1 (DOCX 1294 KB)

## Data Availability

The authors confirm that the data supporting the findings of this study are available within the article and its supplementary materials. Derived data supporting the findings of this study are available from the corresponding author MT on reasonable request.

## Appendix 1: Customer pTx-based static ${B}_{1}^{+}$-shimming

The combined ${B}_{1}^{+}$ field created by the transceiver array is expressed as

1 $$B_{1c}^{ + } \left( {\text{r}} \right) = \,\,\left| {\mathop \sum \limits_{{{\text{k}} = 0}}^{{\text{N}}} {\text{C}}_{{\text{k}}} {\text{s}}_{{\text{k}}}^{ + } \left( {\text{r}} \right)} \right|,$$

where, ${\mathrm{C}}_{\mathrm{k}}=\left|{\mathrm{C}}_{\mathrm{k}}\right|{\mathrm{e}}^{\mathrm{i}{\mathrm{\varphi }}_{\mathrm{k}}}$ are the complex weights of the driving current for channels and ${\mathrm{s}}_{\mathrm{k}}^{+}\left(\mathrm{r}\right)$ are spatial ${B}_{1}^{+}$ maps of the individual array elements. Static pTx ${B}_{1}^{+}$ shimming is performed by manipulation of the transmit vector $\left\{\mathrm{C}\right\}=\left\{{\mathrm{C}}_{1}\cdots {\mathrm{C}}_{\mathrm{N}}\right\}$. Two cost functions based on statistical metrics of ${B}_{1c}^{+}\left(\mathrm{r}\right)$ computed within the volume $\mathrm{\Delta r}$ were defined as

2 $${\mathrm{F}}_{\mathrm{homo}}\left(\mathrm{\Delta r},\left\{\mathrm{C}\right\}\right)=\left(\frac{\mathrm{std}\left({B}_{1c}^{+}\left(\mathrm{r}\right)\right)}{\mathrm{mean}\left({B}_{1c}^{+}\left(\mathrm{r}\right)\right)}\right)\left(\frac{\mathrm{mean}\left({B}_{1c}^{+}\left(\mathrm{r}\right)\right)}{\mathrm{max}\left({B}_{1c}^{+}\left(\mathrm{r}\right)\right)-\mathrm{min}\left({B}_{1c}^{+}\left(\mathrm{r}\right)\right)}\right)^{-1}$$

3 $${\mathrm{F}}_{eff}\left(\mathrm{\Delta r},\left\{\mathrm{C}\right\}\right)=\left(\frac{\mathrm{std}({B}_{1c}^{+}\left(\mathrm{r}\right))}{\mathrm{mean}({B}_{1c}^{+}\left(\mathrm{r}\right))}\right)\mathrm{min}\left({B}_{1c}^{+}\left(\mathrm{r}\right)\right)^{-1}$$

The first function is aimed to minimize the coefficient-of-variation and difference between maximal and minimal value and, thus, to promote the homogeneity of B_1_^+^ distribution is referred as “B_1_^+^-homogeneity”. The second function aimed for the suppression of destructive interferences via maximizing a minimum of B_1_^+^ within optimization volume is referred to as “B_1_^+^-efficiency”.

Additionally, the cost function included the array transmit efficiency ratio of “sum-of-magnitudes” (SOM) and “magnitude-of-sum” (MOS) is defined as

4 $${\mathrm{TX}}_{\mathrm{e}}\left(\mathrm{\Delta r},\left\{\mathrm{C}\right\}\right)=\frac{\left|{\sum }_{\mathrm{k}}^{\mathrm{N}}{\mathrm{C}}_{\mathrm{k}}{\mathrm{s}}_{\mathrm{k}}^{+}\right|}{{\sum }_{\mathrm{k}}^{\mathrm{N}}\left|{\mathrm{C}}_{\mathrm{k}}{\mathrm{s}}_{\mathrm{k}}^{+}\right|}.$$

Finally, the optimization problem is to be solved regarding the transmission vector

5 $$\left\{{\mathrm{C}}_{\mathrm{opt}}\right\}=\mathrm{argmin}\left(\frac{{\mathrm{F}}_{\mathrm{cost}}\left(\mathrm{\Delta r},\left\{\mathrm{C}\right\}\right)}{{\mathrm{TX}}_{\mathrm{e}}\left(\mathrm{\Delta r},\left\{\mathrm{C}\right\}\right)}\right).$$

Problem (5) was solved by the multi-start application of a constrained minimization solver using the MATLAB optimization toolbox and an in-house developed toolbox for RF-shimming [54]. The normalization boundary conditions for the individual components of the vector $\left|{\mathrm{C}}_{\mathrm{k}}\right|\le 1$ were used to ensure limited RFPA power in each channel. The found optimization vectors were normalized as $\Vert {\mathrm{C}}_{\mathrm{opt}}\Vert =1.$ The normalized complex vectors were set to the RFPA using the “patient-specific” mode of the ${B}_{1}^{+}$ adjustment of the scanner.
