# Supplementary material for: High-resolution imaging of the excised porcine heart at a whole-body 7 T MRI system using an 8Tx/16Rx pTx coil
Source: MAGMA. 2023 Apr 7;36(2):279–93. doi: 10.1007/s10334-023-01077-z (PMC10140105; doi:10.1007/s10334-023-01077-z)
Supplement: Supplementary file 1 — Supplementary file1 (DOCX 1294 KB) [file 10334_2023_1077_MOESM1_ESM.docx]

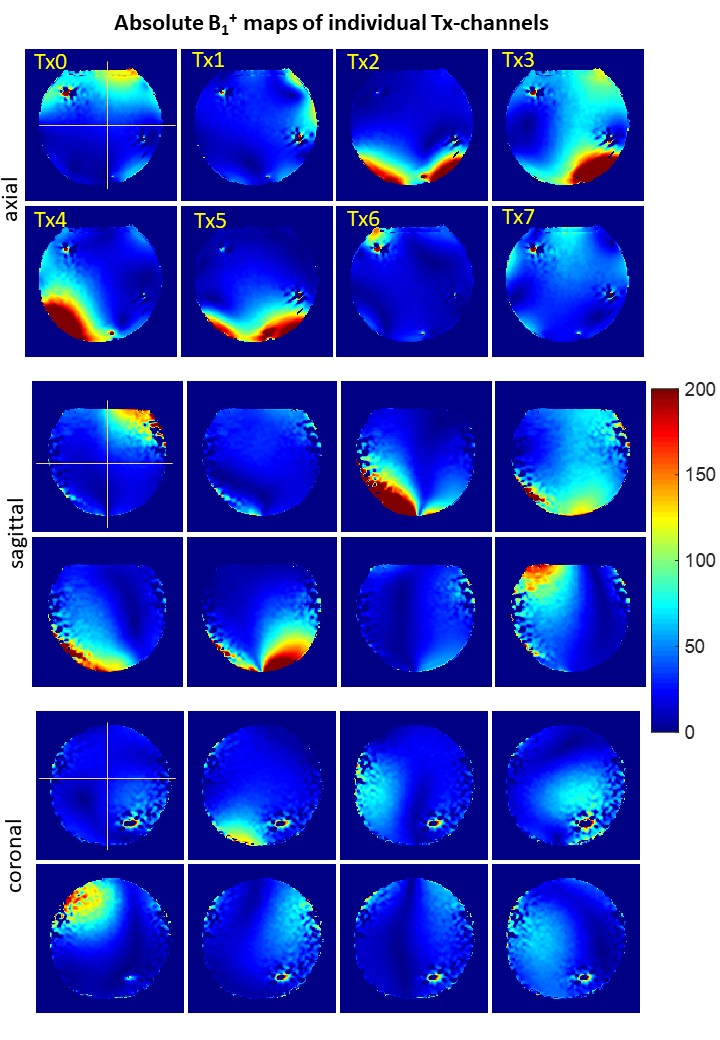


Figure S1. Absolute B_1_+ maps of the individual Tx channels of the new 8Tx/16Rx array. The high similarity of the B_1_^+^ profiles with the relative B_1_^+^ maps (Figure 3) is confirmed. The typically numerous reconstruction artifacts of absolute B_1_^+^ mapping make relative B_1_^+^ maps more convenient for the numerical optimization procedure of pTX-based B_1_^+^ shimming.


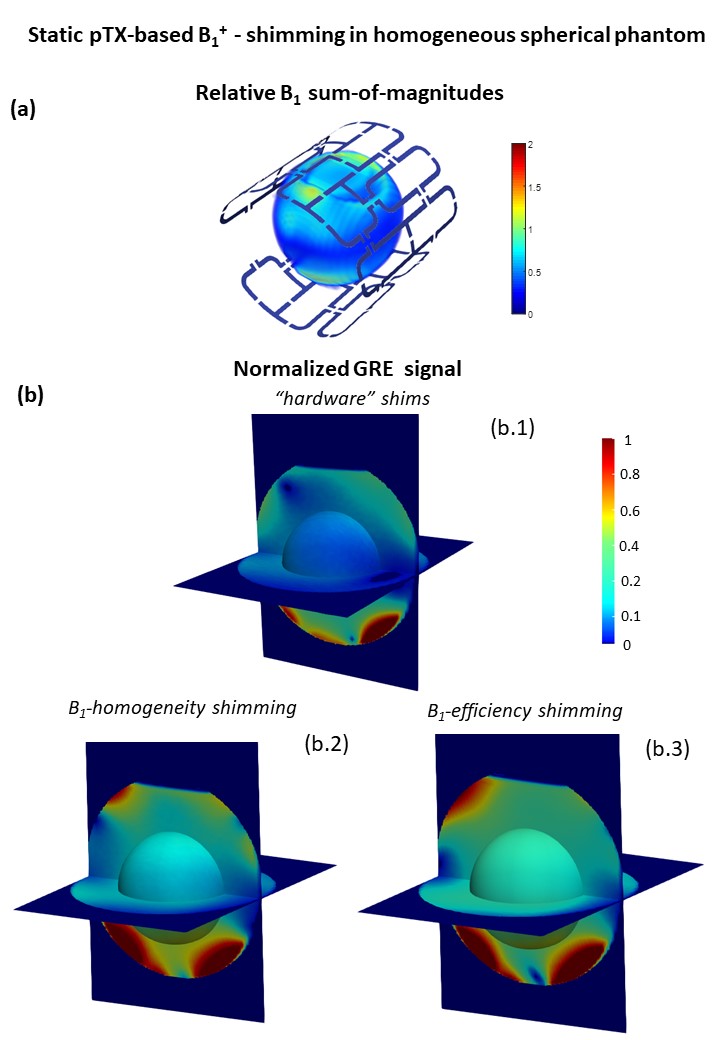
Figure S2. Figure S2. Static pTX-based B_1_^+^ shimming in 10 cm sphere filled with PVP solution. (a) Sum-of-magnitudes of relative B_1_^+^ in all channels with an overlay of the array elements schematic (b) Results of pTX-based static B_1_^+^ shimming. GRE images were acquired with the same set RF-pulse voltage. Normalized signal projections are shown in the central transversal plane and on spherical surface slice b.1: before optimization (default hardware-based shims), b.2: after optimization with „B_1_^+^-homogeneity” cost function, and b.3: after optimization with „B_1_^+^-efficiency ” cost function (see Appendix 1)


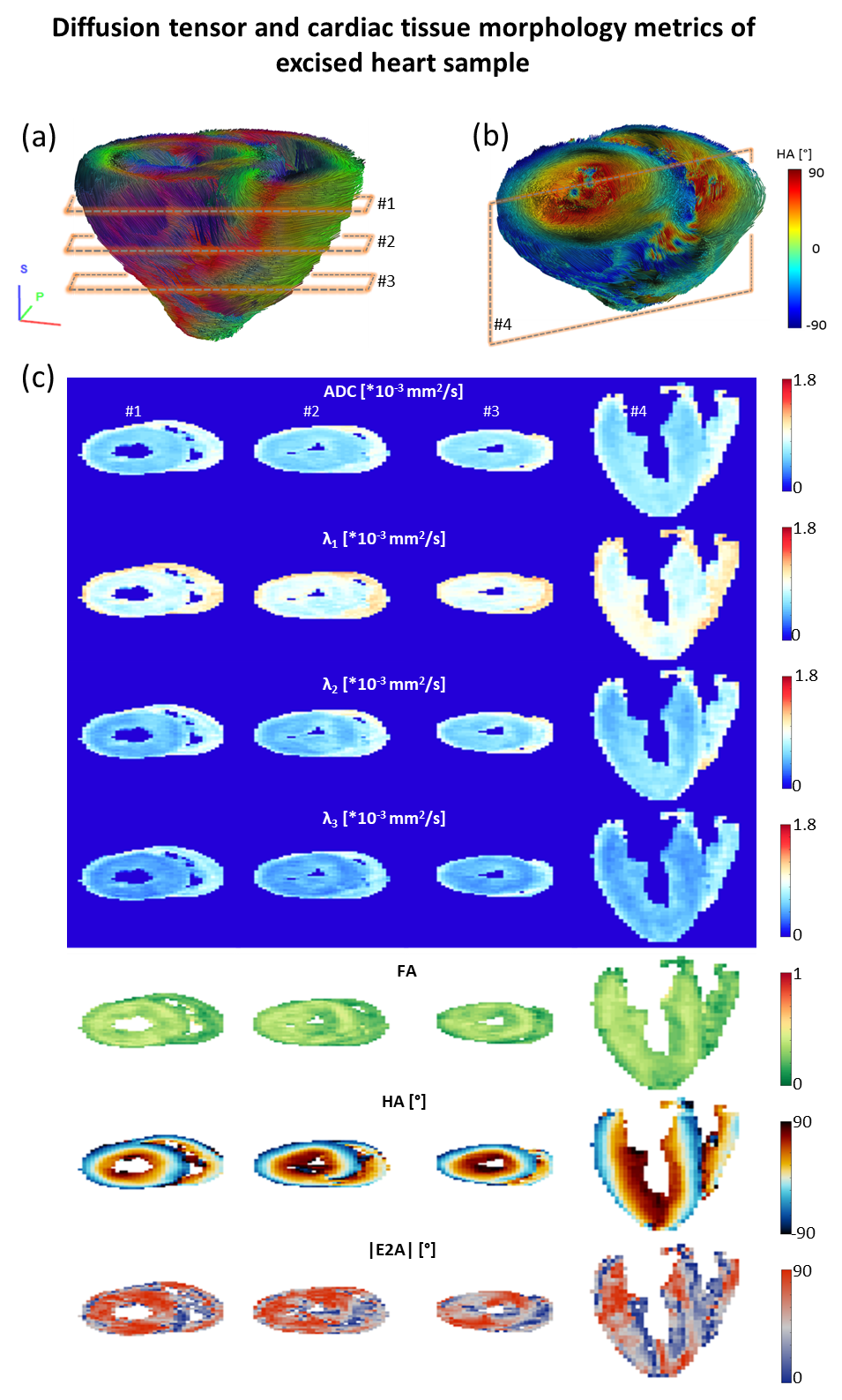
Figure S3: Metrics of cardiac diffusion based on high resolution diffusion tensor imaging of the porcine heart acquired using the dedicated 8Tx/16Rx array. (a) Tractography showing 30000 reconstructed myocyte bundles. Color-coding according to main eigenvector orientation. Respective axes are shown in the coordinate system in the bottom left. Dashed boxes approximate slice positions for short axis images shown below. (b) Tractography showing 30000 reconstructed bundles with adjusted angulation compared to (a). Color-coding according to the local helix angle value. The dashed box approximates the position for the long axis images shown below. (c) Basal, mid-cavity, apical short axis as well as a long axis map, depicting cardiac DTI metrics: apparent diffusion coefficient (ADC), eigenvalues of the diffusion tensor (λ1-λ3), fractional anisotropy (FA), the helix angle (HA) as well as the absolute sheetlet angle (|E2A|).
